# Validation of expert system enhanced deep learning algorithm for automated screening for COVID-Pneumonia on chest X-rays

**DOI:** 10.1038/s41598-021-02003-w

**Published:** 2021-12-01

**Authors:** Prashant Sadashiv Gidde, Shyam Sunder Prasad, Ajay Pratap Singh, Nitin Bhatheja, Satyartha Prakash, Prateek Singh, Aakash Saboo, Rohit Takhar, Salil Gupta, Sumeet Saurav, Raghunandanan M. V., Amritpal Singh, Viren Sardana, Harsh Mahajan, Arjun Kalyanpur, Atanendu Shekhar Mandal, Vidur Mahajan, Anurag Agrawal, Anjali Agrawal, Vasantha Kumar Venugopal, Sanjay Singh, Debasis Dash

**Affiliations:** 1 CSIR-Central Electronics Engineering Research Institute, Pilani, Rajasthan 333031 India; 2 CSIR-Institute of Genomics and Integrative Biology, Mathura Road, New Delhi, 110025 India; 3 Centre for Advanced Research in Imaging, Neurosciences Genomics (CARING), New Delhi, India; 4 Teleradiology Solutions, 12B Sriram Road, Civil Lines, Delhi, 110054 India; 5 Teleradiology Solutions, 7G, Opposite Graphite India, Whitefield, Bangalore, Karnataka 560048 India; 6 Academy of Scientific and Innovative Research (AcSIR), Ghaziabad, 201002 India; 7 Maulana Azad Medical College (MAMC), New Delhi, India

**Keywords:** Biomedical engineering, Translational research

## Abstract

SARS-CoV2 pandemic exposed the limitations of artificial intelligence based medical imaging systems. Earlier in the pandemic, the absence of sufficient training data prevented effective deep learning (DL) solutions for the diagnosis of COVID-19 based on X-Ray data. Here, addressing the lacunae in existing literature and algorithms with the paucity of initial training data; we describe CovBaseAI, an explainable tool using an ensemble of three DL models and an expert decision system (EDS) for COVID-Pneumonia diagnosis, trained entirely on pre-COVID-19 datasets. The performance and explainability of CovBaseAI was primarily validated on two independent datasets. Firstly, 1401 randomly selected CxR from an Indian quarantine center to assess effectiveness in excluding radiological COVID-Pneumonia requiring higher care. Second, curated dataset; 434 RT-PCR positive cases and 471 non-COVID/Normal historical scans, to assess performance in advanced medical settings. CovBaseAI had an accuracy of 87% with a negative predictive value of 98% in the quarantine-center data. However, sensitivity was 0.66–0.90 taking RT-PCR/radiologist opinion as ground truth. This work provides new insights on the usage of EDS with DL methods and the ability of algorithms to confidently predict COVID-Pneumonia while reinforcing the established learning; that benchmarking based on RT-PCR may not serve as reliable ground truth in radiological diagnosis. Such tools can pave the path for multi-modal high throughput detection of COVID-Pneumonia in screening and referral.

## Introduction

SARS-CoV-2 pandemic created a global disruption on an unprecedented scale^1^. Tracing, testing, isolation, treatment is the universally agreed-upon strategy along with the laid standard operating procedures for limiting the spread. But this sequence often faced a bottleneck at the testing stage. Quantitative Reverse Transcription Polymerase Chain Reaction (qRT-PCR), the gold standard, is a specialized test often not available in a timely manner^2^ due to being time-consuming and rapid antigen test having a high false-negative rate. Further, infected patients need an assessment of severity which qRT-PCR does not provide. This led to advocacy of using ubiquitously available resources like Chest X-rays for identifying likely infections while awaiting qRT-PCR results and determining the severity of COVID pneumonia. The concept of causative diagnosis from X-rays might be concerning because the conventional approach of understanding pathologies is based on causality. Most deep learning algorithms on the other hand learn patterns of association by analyzing large volumes of data thereby offering an opportunity to identify associations hitherto unknown or unexplored by medical practitioners. Several studies have demonstrated that deep learning can make causative diagnoses like tuberculosis from X-rays as good as radiologists^3^.

COVID-19 pneumonia (CoV-Pneum) was seen to have characteristic findings on Chest X-rays (CxR); Bilateral peripheral hazy or consolidative opacities were seen; usually without pleural effusion or mediastinal lymphadenopathy^4–7^. These findings overlap with presentations of other atypical or viral pneumonia but are different from the typical bacterial pneumonia with large asymmetric consolidation, air bronchograms, and pleural effusion. Experienced radiologists use such distinctions to determine whether an abnormal CxR represents a high likelihood of CoV-Pneum. Non-pneumonia forms of COVID-19 cannot be diagnosed on CxR, at least by the human eye. Artificial Intelligence (AI) algorithms for COVID-19 prediction on CxR have claimed high performance in determining not only CoV-Pneum but also SARS-CoV-2 infection without any obvious pneumonia on CxR^8–10^. Most of these algorithms are deep learning (DL)-based^11–15^ trained on a limited amount of COVID-19 data that is available, and without clear explainability. This led to three concerns; First, robustness or generalizability is typically low for unexplainable AI, especially when DL systems are trained on smaller datasets. Second, in small datasets when a subset is set aside for validation, apparent algorithm performance is artificially boosted. Last, many datasets do not make clear distinctions between the RT-PCR diagnosis of SARS-CoV-2 infection and the radiological or clinical one of CoV-Pneum. It is *apriori* unlikely that AI tools can detect SARS-CoV-2 infection that has not manifested in the lung, on a CxR. Thus, it seems reasonable to develop explainable AI tools that merge established DL capacity in detecting routine CxR pathology, with a logic-based determination of CoV-Pneum likelihood from nature and spatial distribution of the pathology. Whether such an approach would perform as well as pure DL-based methods is not known.

Here, we report an AI classifier of CoV-Pneum that is composed of an ensemble model consisting of three DL modules and an expert decision system. The DL modules are; pathology classification, lung segmentation, and an opacity detection module which are explainable to the extent of an activation map output. The expert decision system is a rule-based classification system that classifies the X-ray into one of three classes, namely COVID-unlikely, indeterminate, and COVID-likely and is fully explainable as well as modifiable as required based on the pathology in question. We tested this classifier on two test sets that had not been seen by the algorithm. First, a general population dataset of randomly selected CxR from an Indian quarantine center, where SARS-CoV-2 infection status was not known, was used as a testing dataset. To evaluate our proposed method on these CxRs, COVID likelihood marked by a single radiologist was compared against. Second, a curated data set for which SARS-CoV-2 infections status was clearly known and four independent radiologists had classified the probability of CoV-Pneum and marked the lesions. Other than concordance with RT-PCR and radiological diagnosis, we also defined concordance between the bounding boxes of lesions identified by the radiologist and the CovBaseAI algorithm. We found that our approach matches the performance of published DL systems while providing a high degree of explainability. Given the social and medical consequences of being declared “COVID positive”, this appeared to be a better approach. One of the reasons we chose to have an additional expert decision system on top of the three independent DL models was to ensure that no individual model controls the outcome^16^.

## Related work

The advent of deep learning techniques and their applications in the tasks involving classification^17,18^ and object detection^19,20^ has already proven its efficacy which led to the escalation in efforts for the development of deep learning based methods for the screening of Chest X-rays^21^ for SARS-CoV-2. Multiple studies have proposed detection of SARS-CoV-2 based on CT X-ray data^11–15,22,23^. Leveraging CxR imaging for screening of COVID-19 pandemic has several advantages such as portability and accessibility, specifically in areas having inadequacy of resources and areas which are declared as hot spots for viral infection and thus can aid in triaging of affected patients.

As one of the early efforts^22^ proposed a convolutional neural network based approach for screening of COVID-19 patients. They termed it COVID-Net and trained it using the open-source COVIDx dataset^24^. COVID-Net incorporated features such as architectural diversity, long-range connectivity, and a lightweight design pattern for classification among normal, COVID-19, and pneumonia CxRs. COVID-Net reported an accuracy of 93.3% on the COVIDx dataset, a sensitivity of 91% for COVID-19, and a high positive predictive value (PPV) reflecting few false positives and also incorporated explainability features by leveraging GSInquire^25^. In another study proposed by Ozturk et al., DarkCOVIDNet^11^ architecture was presented which was inspired from DarkNet^26^, a proven deep learning architecture for high-speed object detection. DarkCOVIDNet was trained on a dataset of 1125 images comprising of COVID-19(+), Pneumonia, and No-Findings. It resulted in an accuracy of 98.08% and 87.02% for binary and three classes, respectively. Performance of both the architectures^11,22^ discussed above can be significantly improved provided they are trained on a larger dataset and can result in increased generalization. However to overcome the issue of data scarcity Afshar et al.^12^ proposed COVID-CAPS based upon capsule networks^27^ as conventional convolutional neural networks (CNNs) can lose spatial information between image specimens and need substantially larger datasets to train for better performance. COVID-CAPS on the other hand is capable of handling small datasets. COVID-CAPS was trained on a dataset having four labels i.e. Normal, Bacterial, Non-COVID Viral, and COVID-19, and obtained an accuracy of 95.7% and specificity of 95.8% which is further enhanced after incorporating pre-training and transfer learning based on an additional dataset of X-ray images. Many of these models just reported cross-validation accuracy and did not reflect upon the need to test the developed algorithms on independent blind datasets based on both the RT-PCR tests and the radiological findings. Maguolo and Nanni^28^ who have reviewed the recently published literature on CxR based COVID-19 detection and included most of the recently published datasets which had been utilized for deep learning based COVID-19 detection; demonstrated that models could identify the source correctly even after the lung fields are masked with black boxes thereby concluding that most models might be biased and learn to predict features that depend more on the source dataset than the relevant medical condition. A review by Michael Roberts et al.^16^ described the common pitfalls when developing an AI-based method for COVID-19 detection and prognosis. Out of the 2212 papers, they found in the literature, they filtered these papers based on a pre-set criteria to finally review 62 papers. While reviewing these papers, it was observed that none of the methods published are suitable for adoption in a clinical setting due to flaws in their methodology and the inclusion of certain underlying biases. In view of the aforementioned literature, we have tried to comply with most of the recommendations and address their lacunae; such as using standard and curated datasets, not using RT-PCR as the only ground truth, and various methodological considerations along with a robust rule-based system devised by experts themselves, etc.

Arias-Londoño et al.^29^ described how pre-processing improves performance and explainability during detection of COVID-19 from CxRs. They found that segmenting the lungs automatically before detecting COVID-19 improves explainability performance considerably and is useful for deployment in a clinical setting. BS-Net^30^ published by Alberto Signoroni et al. demonstrated an end-to-end framework for a multi-regional score to assess the degree of lung compromise on CxRs. They have curated an annotated dataset of over 4000 CxRs in-house and have built a Deep Learning based method for segmentation, alignment, and COVID-19 severity score calculation. They have divided the lung into six regions and the severity score was calculated for each region. The final output is provided along with super-pixel-based explainability maps. CovBaseAI follows a similar approach by integrating the diagnostic and detection information from deep learning modules with an Expert Decision System to calculate COVID-19 likelihood. For tools to be useful in screening and triaging; explainability and grading of disease is needed. Additionally, there have been several other investigations on COVID-19 detection employing deep learning based methods utilizing CT scans and chest X-ray images^31–33^.

Although several tools and models have been developed and published that claim to detect COVID-19 infection from CxRs, they lack from a perspective of testing outcomes on images from different geographical entities, image quality effects, and performance in comparison to RT-PCR as a gold standard. Hence, in the context of the aforementioned limitations, we went with a non-conventional approach to detect COVID pneumonia and not infection utilizing a set of pre-trained algorithms. Further, we developed an expert derived decision-making system which could harness the power of machine learning along with intellectual learning to be of use in real case scenarios and be utilized for as a solution for clinical triaging in resource-constrained environments. To address this, we propose a robust novel framework comprising coalescence of deep learning architectures and rules devised by radiologists for screening of COVID-19 disease. In the following sections, the proposed methodology is described in detail followed by datasets utilized for training evaluation and the results obtained.

## Methodology

**Figure 1:**
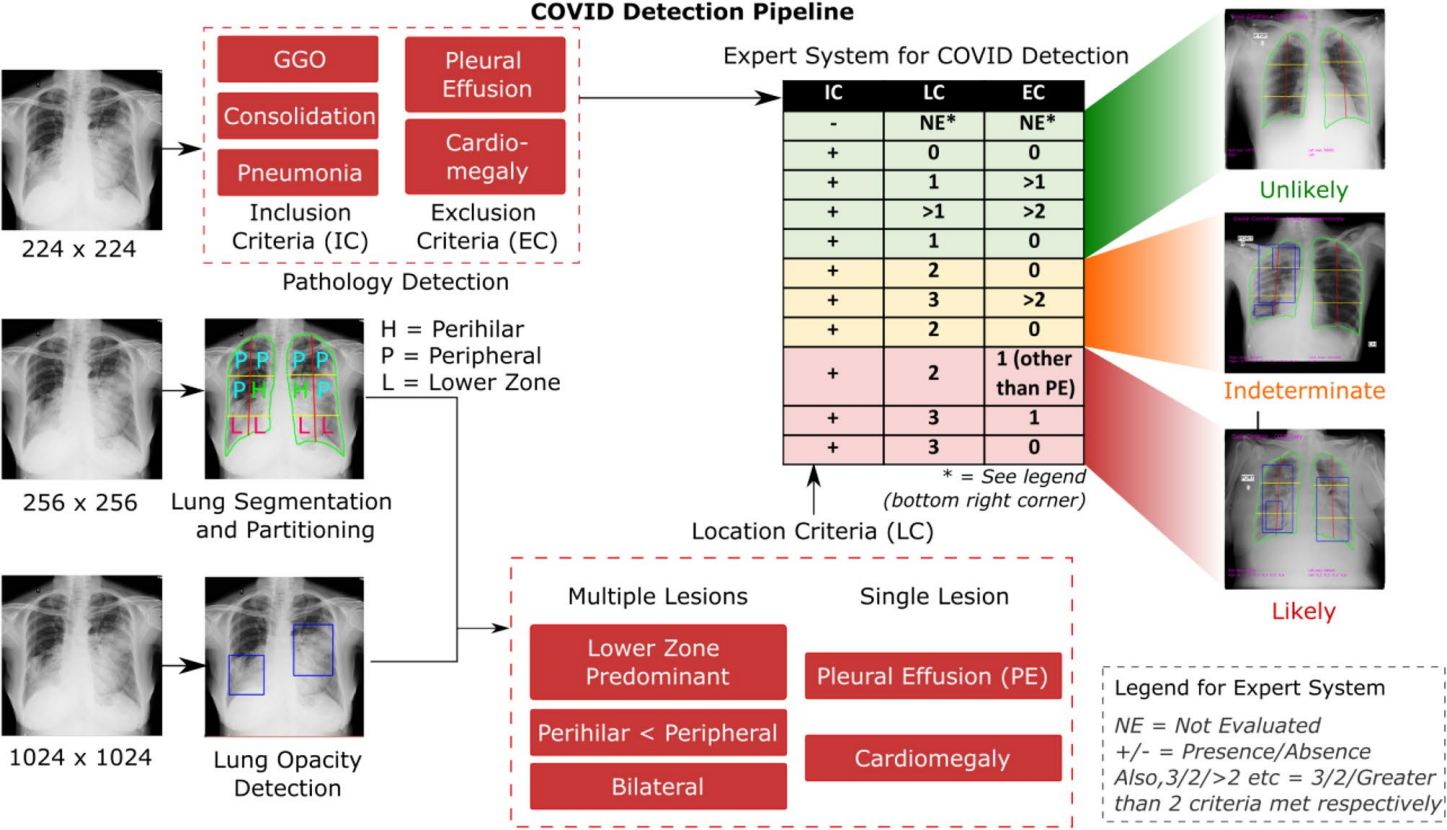
CovBaseAI model architecture for COVID likelihood detection.

The architecture of CovBaseAI is depicted in Fig. 1. It comprises of three deep learning modules responsible for lung segmentation, lung opacity detection, and chest X-ray pathology detection. Each deep learning module is operating independently of the other and has a different input size. The input X-ray image is resized to 256 × 256, 1024 × 1024, and 224 × 224 for lung segmentation, lung opacity detection, and chest X-ray pathology classification module respectively. Lung fields identified as an output of the lung segmentation module are partitioned into different zones and the bounding boxes that are obtained from the opacity detection module are projected onto the original chest X-ray image. The pathology detection module provides the probabilities associated with pathology labels mentioned in the CheXpert dataset^34^. Finally, the outputs of all the deep learning modules are fed into the expert system to assign the COVID likelihood.

### Ethical approvals

The study was duly approved by the Council of Scientific and Industrial Research (CSIR)—Institute of Genomics and Integrative Biology (IGIB) Human Ethics Committee and approval number CSIR-IGIB/IHEC/2020-21/02. The research was performed in accordance with the Indian Council of Medical Research (ICMR) Guidelines on Biomedical and Health Research with Human Participants 2017. Data from IIT Alumni Association and Mahajan Imaging was provided under MoU’s with reference number AA/MOU/RS/080720 and CEERI/IGIB/TRS/MI/052020/01 respectively for research purposes in early times of COVID-19 and was anonymized for all purposes. Due to the retrospective nature of the study, informed consent was waived by the Council of Scientific and Industrial Research (CSIR)—Institute of Genomics and Integrative Biology (IGIB) Human Ethics Committee.

### Lung segmentation

The lung segmentation module is a fully connected network adapted as a modification of U-Net^35^; a proven convolutional network for biomedical image segmentation. The CxR image was resized to 256 × 256 and provided as input to the lung segmentation module to obtain the output lung mask. U-Net being a convolutional neural network is open to layer-wise modifications and blocks of various other neural networks to enhance the segmentation output. In the U-Net architecture, we replaced the encoder part with the VGG16^36^ network which is pre-trained on the ImageNet^37^ dataset and thus substantially reduces training weight convergence time and aids to prevent over-fitting^38^ as has been demonstrated previously with Chest X-rays^39^. For training the segmentation module, a batch size of 4 was used, the learning rate was kept at 0.001, the loss function used was BCEWithLogitsLoss, Adam was used as the optimizer and it was trained for 30 epochs.

### Lung opacity detection

Lung opacity detection was performed using a Faster R-CNN^19^ architecture utilizing the VGG16 network as the backbone for feature extraction in Faster R-CNN. It is a two-stage object detection network that offers high accuracy as compared to a single-stage network. It is a merger of the region proposal network (RPN) and the Fast R-CNN^40^ method. In the first stage; object-like proposals are generated, the second stage focuses on recognition of these proposals. Input to the Lung opacity module is the CxR image which is resized to 1024 × 1024 and the bounding boxes surrounding opacity regions are obtained as output. The hyper-parameters for opacity detection module were: for the two components in RPN, softmax classifier was used for determining the loss in score generation of each region predicted by the classification network. Smooth L1 loss was deployed to compute the loss in the regression layer, batch size was kept as 8, Adam was used as the optimizer, the learning rate was set to 0.001 and it was trained for 100 epochs.

### Pathology detection on chest X-rays

Pathology detection on chest X-rays was performed using DenseNet-201^17^ as a backbone network. The input CxR image was resized to 224 × 224 using the linear interpolation^41^ technique and was initialized with pre-trained weights of the ImageNet dataset using a transfer learning approach similar to the technique used in Pham et al.^42^. The pathology detection module outputs the probability corresponding to each pathology which is taken into consideration by the rule-based COVID-19 likelihood detector to give the final prediction. In the CheXpert dataset, three labels are given corresponding to every image for each pathology i.e. positive, negative, and uncertain. While training the pathology detection module; uncertain labelled images were considered as negative according to the “U-Zero approach” mentioned in the CheXpert documentations^34^ and only frontal CxRs were taken into account. The CheXpert validation dataset^34^ does not contain uncertain labels, only positive and negative labels. In our work, uncertain labels were taken as negative and utilized because; when the U-Zero approach is followed as per literature^34^; the best AUC is achieved for consolidation specifically. Consolidation is one of the most important markers for the detection of pneumonia and is a major manifested anomaly. Hyper-parameters for training the pathology detection module were: a batch size of 32 was used, the learning rate was kept at 0.001, Adam was used as an optimizer, the Sigmoid classifier was employed and it was trained for 20 epochs.

### Lung opacity detection with lung partition

Lung mask coordinates obtained from the lung segmentation module aids us in identifying the left lung and right lung on the input CxR which are then partitioned into six different regions to localize the perihilar, peripheral, middle, upper, and lower zone segments of the two lungs. This zonal classification roughly corresponds to the locations of anterior ribs^43^. The lung parenchyma overlying the upper two anterior ribs corresponds to the upper zone, the next two ribs to the mid zone, and the lower anterior ribs correspond to the lower zone. Partitioned lung along with the bounding box coordinates obtained from the opacity detection stage was developed to assist the rule-based expert system for COVID-19 classification to predict COVID-19 likelihood.

**Figure 2:**
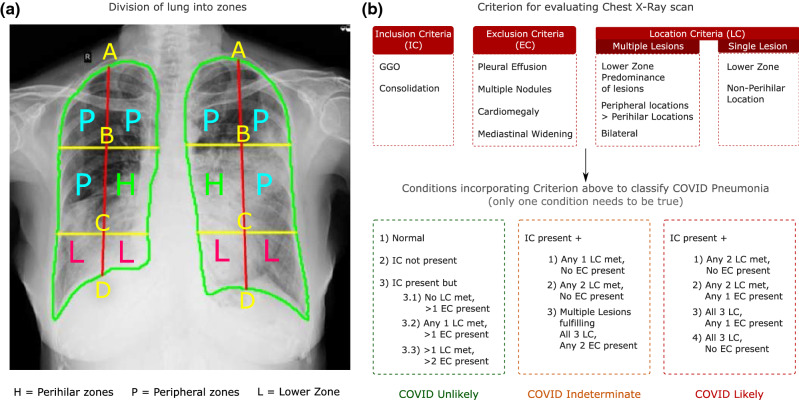
(**a**) Lung partitioning framework. (**b**) Rule-based expert decision system devised by radiologists.

As shown in Fig. 2a after the lungs segmentation module detects the left lung and right lung separately, the uppermost (A) and lowermost (D) coordinate of each lung are connected. We then divided the line A-D into two equal partitions to obtain points B and C and drew a horizontal line on B and C thus portioning each lung into six zones. This was devised in view of the observed distribution of lesions in COVID pneumonia and was not meant to alter the known anatomy and lobular classification.

### Rule-based expert decision system for COVID classification

The rules for classification were framed by a consensus among four radiologists with more than ten years of experience in thoracic radiology. The frequency of findings on chest imaging studies including Chest X-rays and CT scans reported by several researchers were considered in establishing the consensus rules^4,5,44^.

The rules were framed based on three broad sets of criteria: Lesion inclusion criteria, Lesion exclusion criteria, and Location criteria for lesions and pathology identified. Figure 2b delineates the rule-based inference logic utilized for defining COVID-19 likelihood. In the available literature, the algorithms have been trained to identify subtle features by neural networks which could help differentiate COVID images from Non-COVID images. We have here demonstrated an amalgamation of learning networks with the experience of radiologists to have expert systems which help define the COVID likelihood.

### Validation

To evaluate the performance of the CovBaseAI model, we used two datasets. First, 1401 randomly selected CxR from an Indian quarantine center to assess effectiveness in excluding radiologic Cov-Pneum that may require higher care. Second, a curated data set with 434 RT-PCR positive cases of varying levels of severity and 471 historical scans containing normal studies and non-COVID pathologies, to assess performance in advanced medical settings. We compared the outputs of the model against the ground truth. Since radiological and RT-PCR ground truth vary and each has a significant error associated with it, this was done in multiple different ways as described in the dataset section and results. Generally, we prioritized radiological ground truth since that is the most relevant use case scenario at the current state of technology.

## Datasets

### Training and validation datasets

#### Lung segmentation

For training and validation of lung segmentation module, a subset of RSNA Pneumonia Detection Challenge dataset^45^ was used which is similar to https://github.com/limingwu8/Lung-Segmentation. It consists of 1000 CxR images along with their corresponding binary mask. Out of these 1000 CxRs, 900 CxRs were used for training and 100 CxRs were used for validation. The images and their corresponding binary mask were originally of size 1024 × 1024 pixels but they were resized to 256 × 256 pixels as training the model using the original size required high RAM consumption.

#### Lung opacity detection

Lung Opacity detection discussed in the methodology section was performed using RSNA pneumonia detection challenge dataset^45^ to identify and localize pneumonia. This dataset consists of 26,684 images of three different classes; Normal, Lung Opacity, and No Lung Opacity/Not Normal. The Lung Opacity class consists of 6012 images. The Normal Class consists of 8851 images and the No Lung Opacity/Not Normal class consists of 11,821 images in DICOM format. For Lung Opacity Class, coordinates in the form of X-min, Y-min, height, and width for lung opacity are provided. The size of each image is 1024 × 1024 pixels and the Lung Opacity class contains 9555 coordinates of lung opacities in 6012 images. The lung opacity detection module was validated on 1012 test X-ray images from the RSNA pneumonia detection challenge dataset.

#### Pathology detection on chest X-rays

Pathology detection on chest X-rays was carried out using the CheXpert^34^ dataset which was released by the Stanford ML group in 2019 as a competition for automated chest X-ray interpretation. CheXpert dataset consists of the following pathologies: Lung Lesion, Edema, Consolidation, No Findings, Atelectasis, Pneumothorax, Pleural Effusion, Pneumonia, Pleural Others, Cardiomegaly, Enlarged Cardiomediastinum, Lung Opacity, Fracture, and Support Devices. Chexpert dataset originally consisted of 234 (AP View/ PA View/ Side View) validation X-ray images out of which only 202 (AP View/ PA View) frontal X-rays were selected as all the Deep Learning modules were trained only on frontal X-rays. The pathology detection module requires 224 × 224 pixels as input size so we resized the original image to 224 × 224 pixels. In the CheXpert dataset three labels were given corresponding to every image for each pathology i.e. positive, negative, and uncertain. The labels of the CheXpert dataset were used as mentioned in Pathology detection under Methodology section.

### Independent testing datasets

As mentioned in “Introduction” section, to avoid bias based on datasets and preventing the algorithm from learning any biased classification, we carefully choose to test the algorithm on three different types of datasets which were standard datasets developed from pre-COVID times, COVID times, and based on RT-PCR results^16^.

To determine usefulness as a screening and triage tool, we used 1401 CxRs (IITAC1.4K) randomly selected from over 8500 CxRs acquired from the NSCI Dome initiative at Mumbai (an Indian quarantine center). SARS-CoV-2 infection status was not known for this dataset, but this reflects an actual mix of cases likely to present to quarantine centers where a determination has to be made regarding patients that can be safely kept in general isolation and those that may need medical care. These 1401 CxRs were carefully annotated by an experienced radiologist for the COVID likelihood. This dataset is composed of 135 COVID likely and 1266 COVID unlikely CxRs. (available at http://covbase4all.igib.res.in).

**Figure 3:**
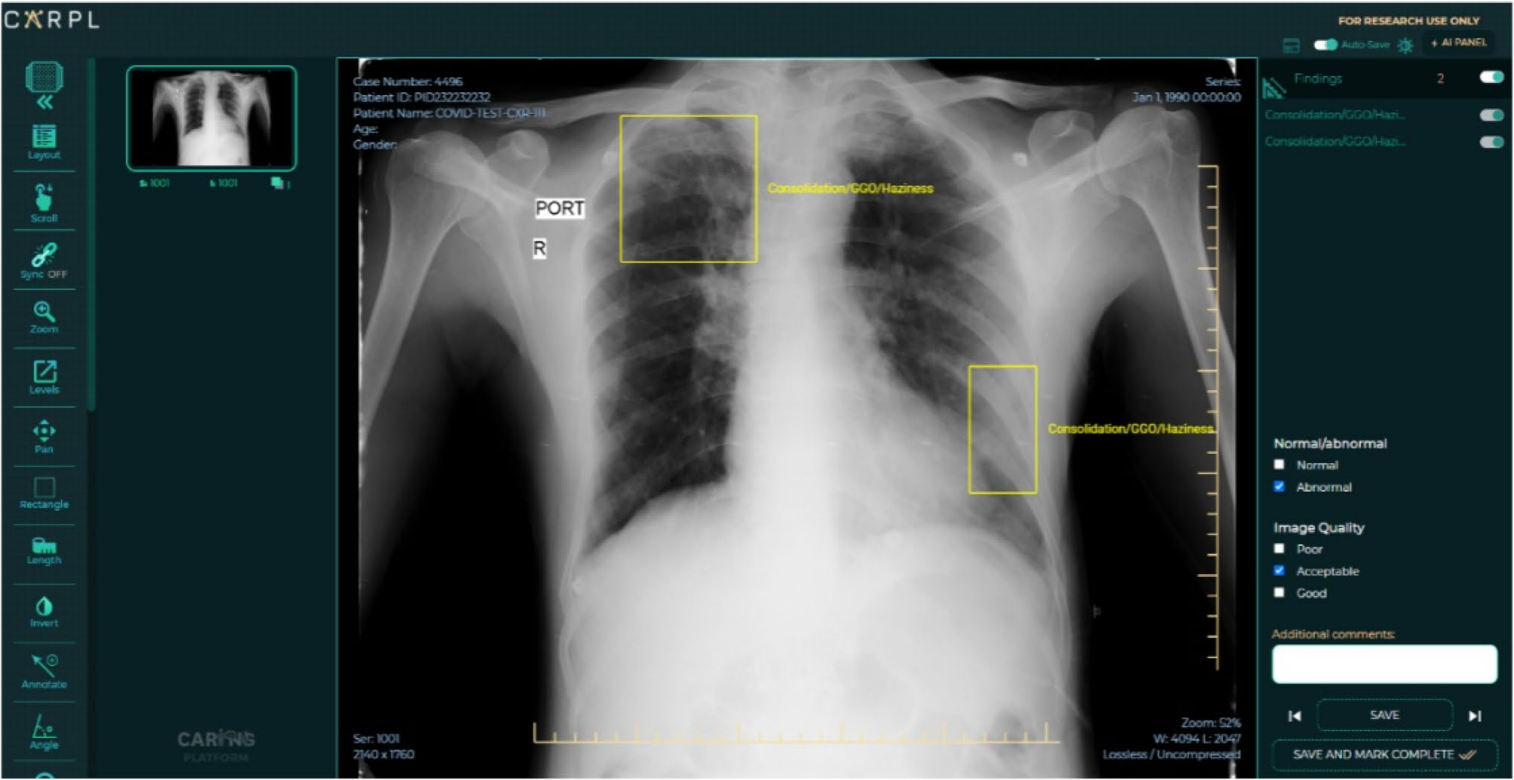
A snippet of the CARPL platform used for annotation and validation. *(Image courtesy: CARING, India).*

Furthermore, the CovBaseAI model can also be used as a diagnostic tool for SARS-CoV-2 infection or confirmed COVID pneumonia, we used a testing dataset which comprised of a total of 905 CxRs acquired from Mahajan Imaging, Delhi. These 905 CxRs comprised of 434 RT-PCR positive scans and 471 historical scans that were acquired before the worldwide outbreak of COVID-19. The X-rays were annotated with the consensus of four senior radiologists on the CARPL platform provided by the Centre for Advanced Research in Imaging, Neuroscience, and Genomics (CARING), India for the presence or absence of consolidation at the study level. A snippet of the CARPL platform used for annotation and testing is depicted in Fig. 3. The presence or absence of Pleural Effusion, Atelectasis, Cardiomegaly, Fibrosis, Mediastinal Widening, Nodule, Pleural Effusion, and Pneumothorax were also recorded. The CovBaseAI model was tested on this set of 905 CxRs using the following three combinations of test scans:

1. *Pneumonia Detector (PD1K) :* Prediction output of the CovBaseAI model was compared against pneumonia/consolidation label annotated by radiologists instead of their RT-PCR status. PD1K consists of 484 pneumonia/consolidation + ve and 421 pneumonia/consolidation –ve labels.
2. *COVID Infection Detector (CID1K) :* Prediction output of the CovBaseAI model was compared against the RT-PCR results for testing its ability to detect COVID infection. CID1K consists of 434 RT-PCR +ve and 471 RT-PCR −ve labels.
3. *COVID Pneumonia Detector (CPD600) :* All cases with RT-PCR positive and radiologist annotated pneumonia/consolidation positive cases were taken as a positive class [336 cases] and all RT-PCR negative and consolidation negative cases [323 cases] were taken as negative class.

Additionally, the CovBaseAI model was also tested on a test dataset as mentioned in Wang et al.^22^. The aforementioned test dataset is referred to as COVIDx1K in the manuscript. The COVIDx1K dataset can be found at (https://github.com/ddlab-igib/COVID-Net/blob/master/docs/COVIDx.md). COVIDx1K comprises of 885 normal and 100 COVID-19 CxRs (test images with pneumonia labels were not considered in the COVIDx1K dataset).

## Results

The segmentation algorithm of lung mask detection was validated using fivefold cross-validation on 100 chest X-rays, from the pool of 1000 X-rays from the RSNA Pneumonia Detection Challenge dataset. We obtained 0.90 as the average Jaccard similarity index^46^.

**Figure 4:**
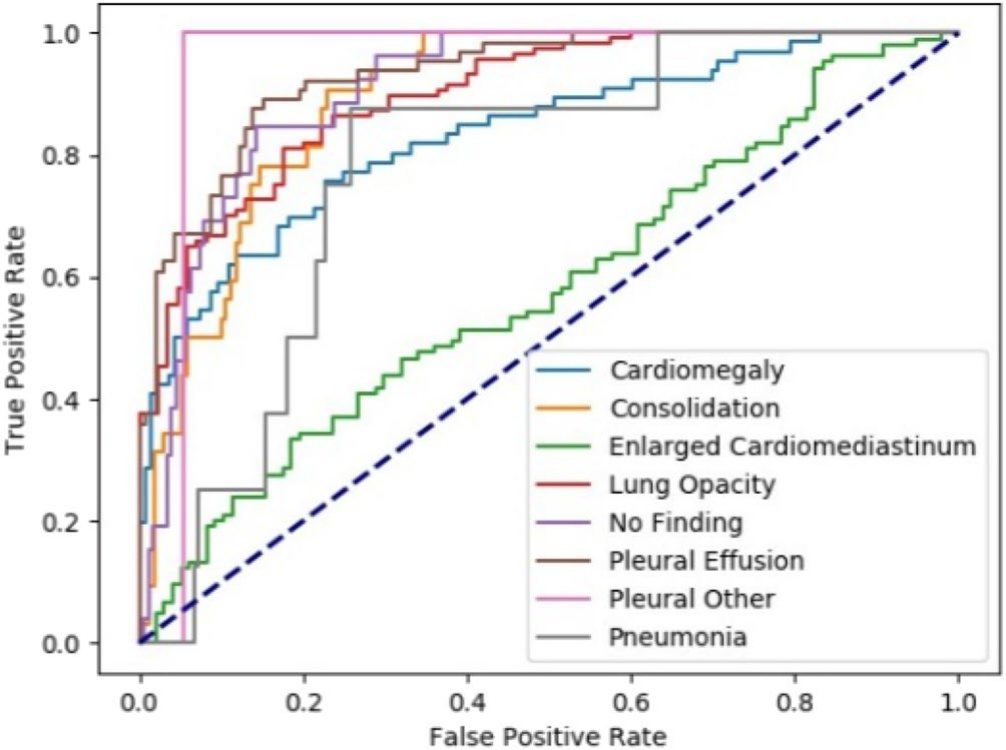
ROC curves of our model for different pathologies on CheXpert validation dataset.

The lung opacity detection module was validated on 1012 test X-ray images from the RSNA Kaggle dataset. The exactness of object detection is usually well determined by mAP (Mean Average Precision)^47^, for opacity detection mAP of 0.34 is achieved. Based upon the pathology detection module’s classification probability scores corresponding to pathologies included in the CheXpert dataset, the AUC’s (Area under the ROC Curve) for Cardiomegaly, Consolidation, Lung Opacity, No Findings, Pleural Effusion and Pneumonia was 0.83, 0.90, 0.90, 0.91, 0.93, 0.78 respectively. The ROC curves for the pathology detection module for different pathologies on the CheXpert validation set have been depicted in Fig. 4.

**Figure 5:**
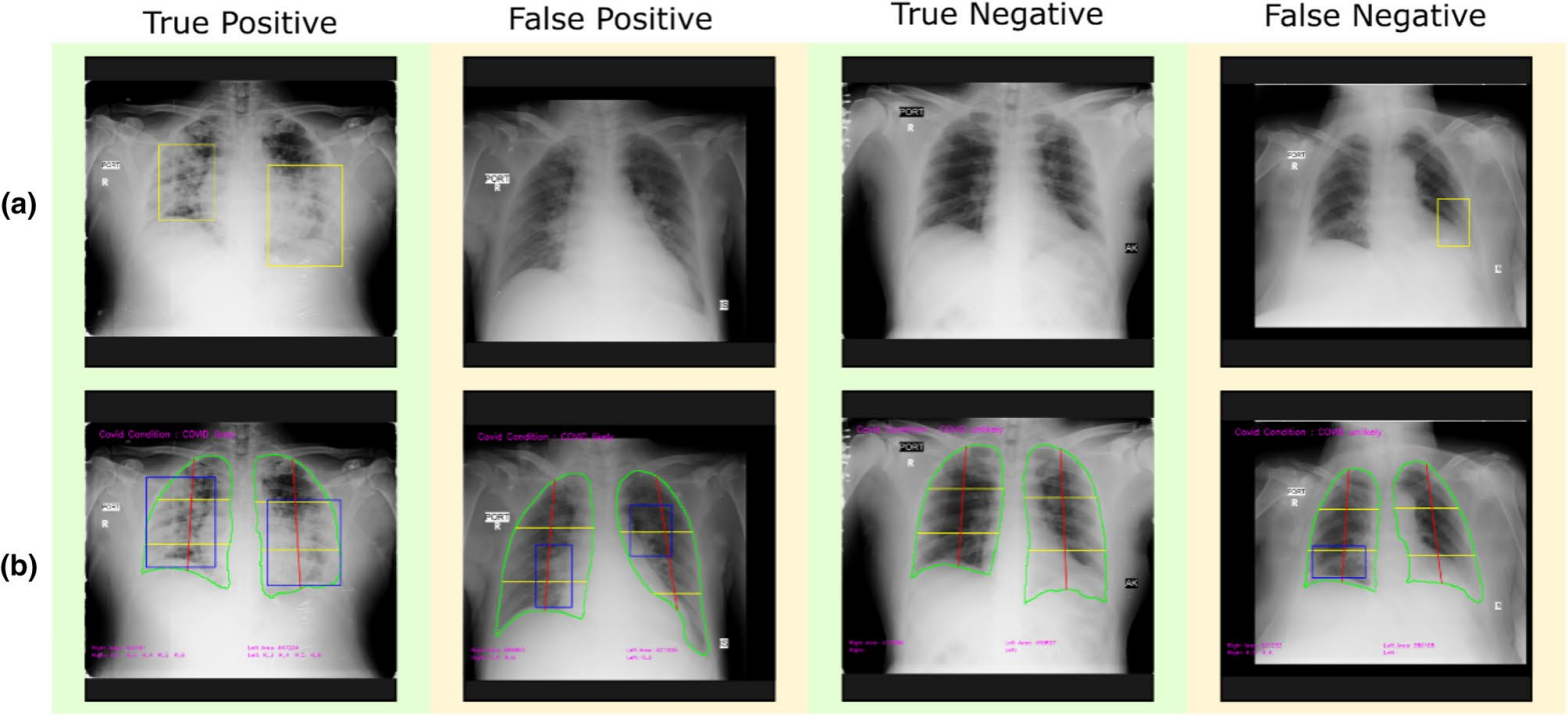
Samples of true positive, false positive, true negative, and false negative from independent validation set of 905 CxRs (**a**) Ground truth CxR (**b**) CovBaseAI inferencing result.

**Figure 6:**
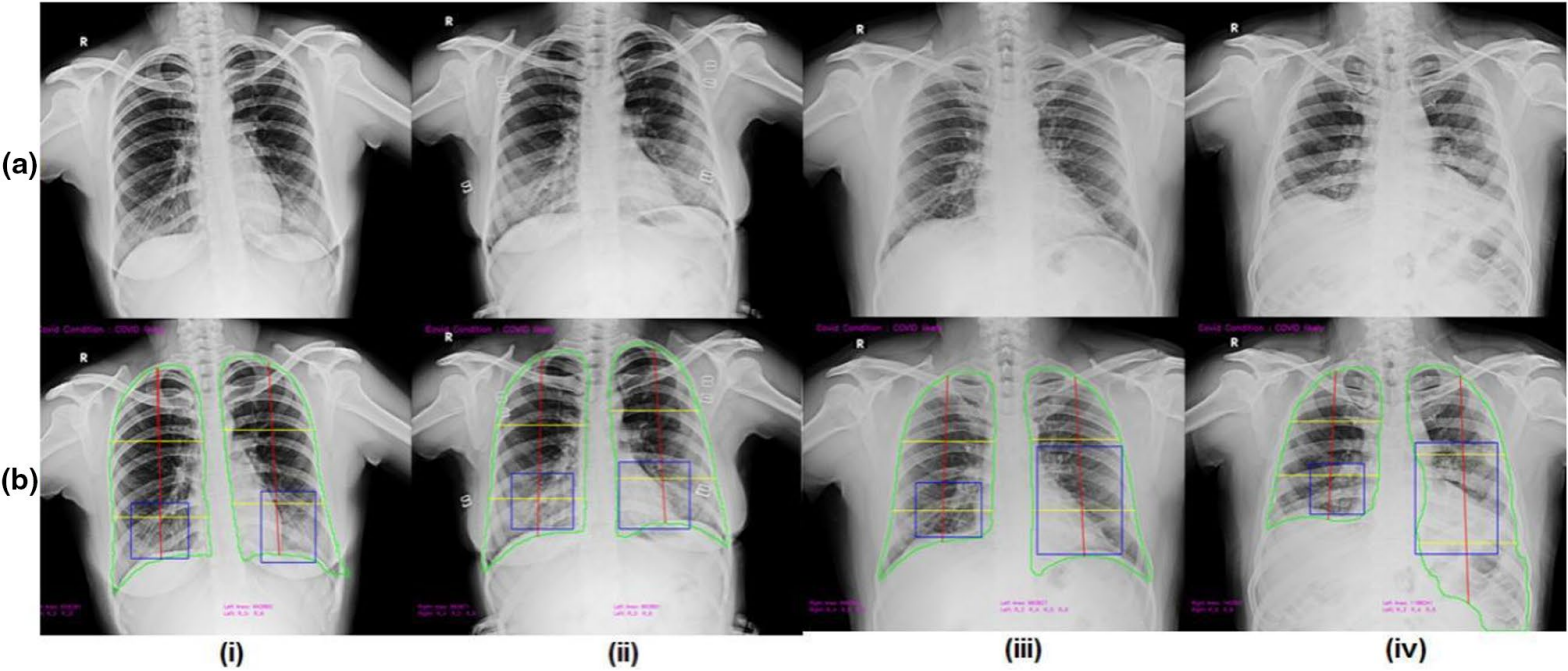
Samples of false-positive from IITAC1.4K dataset (**a**) Ground truth CxR (**b**) CovBaseAI inferencing result.

Table 1 depicts the performance metrics of the CovBaseAI model on different independent validation datasets. Since the ground truth available for the validation dataset was either positive or negative (i.e. RT-PCR +ve/−ve or consolidation +ve/−ve) and the output of the CovBaseAI model is given in three classes (COVID likely, COVID indeterminate and COVID unlikely), the COVID indeterminate class was merged with COVID likely class for calculating performance metrics such as Sensitivity, Specificity, Accuracy, Negative Predictive Value (NPV), etc. Figure 5 depicts the sample of True positive, False positive, True Negative, and False Negative obtained by the CovBaseAI model. Validation studies corresponding to the CovBaseAI model were done using CARPL. Figure 6 shows bounding boxes of representative false-positive images from IITAC1.4K data that were read as normal by the radiologist. On review, the findings inside these bounding boxes were prominent bronchovascular markings, a common finding in the Indian subcontinent, with no clinical significance. Table 2 shows the concordance of bounding boxes (mAP) between lesions identified by AI and radiologists. In 905 CxRs (434 RT-PCR +ve and 471 historical scans) from Independent Validation datasets, read by four radiologists, intersections between AI-human pairs are like human–human pairs. Further, in the case of the IITAC1.4k dataset, which is read by a single radiologist, the majority of the time the bounding box of AI and radiologist had an intersection. Thus, the determination of COVID-19 pneumonia in our model is based on the same parts of the CxR as marked by the expert radiologist. Therefore, the explainability can be considered to be high.

**Table 1:** Performance metrics of CovBaseAI model on independent testing datasets.

Datasets	Sensitivity	Specificity	PPV	NPV	F1 score	Accuracy	MCC	AUC
IITAC1.4K	0.90	0.86	0.41	0.98	0.57	0.87	0.56	0.88
PD1K	0.84	0.81	0.83	0.81	0.84	0.82	0.65	0.89
CID1K	0.66	0.57	0.59	0.64	0.62	0.61	0.23	0.63
CPD600	0.83	0.77	0.79	0.81	0.81	0.80	0.60	0.83
COVIDx1K	0.78	0.97	0.79	0.97	0.78	0.95	0.76	0.89

*PPV* positive predictive value, *NPV* negative predictive value, *MCC* Matthews correlation coefficient, *PD1K* pneumonia detector, *CID1K* COVID infection detector, *CPD600* COVID pneumonia detector.

**Table 2:** Bounding box analysis on 905 CxRs (434 RT-PCR +ve and 471 historical scans) from independent validation datasets.

		Prediction box				
		CovBaseAI (mAP)	RAD 1 (mAP)	RAD 2 (mAP)	RAD 3 (mAP)	RAD 4 (mAP)
**Ground truth**	RAD 1	0.78	1	0.79	0.54	0.79
	RAD 2	0.73	0.80	1	0.51	0.75
	RAD 3	0.43	0.54	0.50	1	0.46
	RAD 4	0.81	0.81	0.75	0.47	1

*RAD* radiologist, *mAP* mean average precision.

To understand the relative performance of our model versus a publicly available COVID-19 CxR algorithm, COVID-CAPS that has reported high-performance characteristics on a subset of data used in Wang et al.^22^, we deployed COVID-CAPS on the Indian dataset. The sensitivity and specificity were only 33% and 67% on IITAC1.4K data that represents an actual use case scenario. The accuracy was below chance (50%) on the CPD600 dataset, which is more challenging. This illustrates the difficulty in the portability of DL solutions to other regions. In contrast, CovBaseAI performed well across datasets, with higher specificity on a western dataset (COVIDx1K) than Indian ones, which reflects the fact that the DL components had never seen Indian data.

## Discussion

There is a great need for AI solutions that have explainability and an assurance of a minimum performance when exposed to data they have not been trained on. While COVID-19 has brought out this need globally, not discriminating between the developed and developing world, the fact of the matter is that this problem has been around for a long time. Here, we have addressed the problem of diagnosing COVID-19 pneumonia in Indian CxR without training on either COVID-19 or Indian CxR data, by using an explainable system driven by logic and expert opinion. While the solution has useful performance characteristics (see Table 1) across Indian (IITAC1.4K, PD1K, CPD600) and western (COVIDx1K) datasets, with high explainability, the insights lie in the differences. In particular, 97% specificity on a western dataset, but 86% specificity on a comparable Indian dataset is a notable comparison. Therefore, our algorithm provides a viable method that combines a radiologist-created EDS fed by three Deep Learning algorithms—the Lung Segmentation module, the Lung Opacity detection module, and the Pathology detection module using Chest X-Rays.

To avoid any bias due to non-relevant areas of the Chest X-Rays, we overlapped the segmented lung lobes prior to EDS similar to Arias-Londono et al.^29^. Using this methodology, we were able to remove diagnostically non-significant features from the deep learning models. We also divided the lungs to provide zone-based inputs to the EDS similar to BS-Net^30^ which also divides the lungs into the zones before feeding into the prediction algorithms. While our lung zones can correspond to lung anatomical structures according to radiologists, our divisions were not anatomically inspired, unlike BS-Net. A pure DL algorithm that reportedly performed well on a subset of the western data, had only 67% specificity on the quarantine center dataset, which makes it unsuited for clinical use in India. COVID-CAPS^12^ used publicly available Western datasets to train DL models for discriminating between bacterial, normal, non-viral COVID-19 and COVID-19. Using the same model on Indian datasets, we found a significant drop in performance compared to published results (33% sensitivity and 67% specificity on IITAC1.4K). This highlights the significant issue of “dirty lungs” in the Indian subcontinent compared to Western datasets thereby needing specialized training datasets. Therefore, similar to recommendations by Roberts et al.^16^, we utilized curated standardized datasets independent from the training datasets for testing the performance of the algorithm. We found that RT-PCR may not be a reliable method for benchmarking COVID-Pneumonia as suggested by Roberts et al.^16^. Given this learning, we took radiologist labels as ground truth for the development of testing datasets for our model.

Most AI solutions, large datasets for training, quality annotations, have come from the developed world. Yet, it is well known that even something as simple as CxRs looks different in regions with high ambient pollution, exposure to dirty fuel, endemic tuberculosis, low body mass, and other differences in lifestyle^48^. It is well known in the radiology community that this well-described “dirty lungs” appearance with prominent bronchovascular markings can easily be confused with abnormal. AI systems, however, are likely to give false positives when exposed to such images, if not previously exposed during training (see Fig. 6 for examples). Such false positives have been part of abstracts and anecdotal experiences^49^ but publication bias against failures has led to a situation where AI solutions for interpreting CxR are working very well in published manuscripts, without similar enthusiasm in actual practice.

The only long-term solution is to have high-quality annotated images from Low-to-Middle-Income countries (LMIC) regions, via global initiatives. In the short term, the use of human logic filters can help in keeping the specificity high enough for clinical use. In our results, the specificity was 97% on a western dataset and 86% for India quarantine center data. Notably, the human logic filters did not substantially compromise sensitivity, which was in the 80–90% zone for COVID-pneumonia, falling to 66% for COVID infection. While it does not seem possible, using our approach, to detect SARS CoV2 infection, without pneumonia, this likely reflects a fundamental lack of sensitivity of CxR rather than a flaw in the algorithm and is consistent with WHO guidelines that do not recommend imaging for infection status determination^50^.

This is one of the limitations of our algorithm and is coherent with the existing literature for not utilizing X-ray based imaging as a diagnostic tool for SARS-CoV2 infection. Another limitation of our work was that training was performed using pre-COVID datasets. This could be argued in favour of the fact that without using a conventional approach, we went with a non-conventional expert-derived rule-based system to achieve a specificity of 86% on Indian datasets. However, COVID datasets could potentially be utilized in the future for training with rule-based systems to enhance accuracy in outcomes. Yet another limitation of the said algorithm is outcome performance on geographically different datasets. This is primarily attributable to the training part with limited data and could be addressed with the use of annotated data from different geographical regions in the future.

The performance characteristics across all the COVID pneumonia datasets are in the clinically useful range and AUC was well above 0.8, the usual threshold at which diagnostic tools are considered useful. Further, the explainability was high (Table 2) and led to an understanding of the underlying issues (Fig. 6), which makes further improvements possible. Such work is now underway and will hopefully lead to more usable AI systems for the Indian subcontinent and similar global regions.

## Conclusion

Several Deep learning architectures have been proposed for COVID-19 detection from CxRs. Most of these methods used limited COVID-19 datasets for training purposes. Models trained on these limited datasets are not ready for real-world usage without training on a large variety of regional datasets to accommodate local variations of data acquisition hardware, population-specific differences, and temporal disease manifestation. For physicians to be able to use AI solutions for screening or triaging, the tools should provide robust detection results, across global data, with explainable findings and grading of severity, but requiring minimal amounts of new data so that solutions can be rapidly developed and deployed. Such an all-inclusive tool is yet to be developed for COVID-19. However, CovBaseAI is a useful step in that direction. We have developed and demonstrated an algorithm as an ensemble of three DL-based models under an umbrella of an expert derived rule-based decision system to detect COVID Pneumonia on chest X-Ray images. The algorithm while addressing fallacies in the available literature and having potential usage in screening and triaging has reinforced the learning that RT-PCR could not reliably be used as ground truth for such algorithms and there is a need for global curated and structured datasets which could be utilized for training of these algorithms to have a wide and universal applicability in clinical scenarios.

## Data Availability

IITAC1.4K is available at http://covbase4all.igib.res.in. COVIDx1K is available at https://github.com/ddlab-igib/COVID-Net/blob/master/docs/COVIDx.md. The other test datasets generated (PD1K, CID1K, CPD600) for this study may be available for research purposes from the corresponding author upon reasonable request. Public datasets used for training and validation in the current study are available at their respective source locations (CheXpert^34^, RSNA Pneumonia Dataset^45^).
